# Observation of choking reaction and other related indexes in elderly painless fiberoptic bronchoscopy with transnasal high-flow humidification oxygen therapy

**DOI:** 10.1515/med-2024-1064

**Published:** 2024-11-21

**Authors:** Yankun Feng, Zhijun Chen, Jiafang Wang

**Affiliations:** Department of Anesthesia, Wuhan No. 1 Hospital, Wuhan, 430022, Hubei, China; Department of Anesthesia, Wuhan No. 1 Hospital, No. 215 Zhongshan Avenue, Qiaokou District, Wuhan, 430022, Hubei, China

**Keywords:** fiberoptic bronchoscope, transnasal high flow, oxygen therapy, elderly patients

## Abstract

**Objective:**

The aim of this study was to observe the effect of nasal high-flow humidification oxygen therapy on choking reactions and related respiratory and hemodynamic effects in elderly patients undergoing fiberoptic bronchoscopy.

**Methods:**

A total of 126 elderly patients aged 65–80 years who underwent painless fiberoptic bronchoscopy from March 2021 to December 2021 were randomly divided into two groups.

**Results:**

The pulse oxygen saturation at T1 and T2 time points in the experimental group was higher than that in the control group (*P* < 0.05), and the average arterial pressure was slightly lower than that in the control group, but there was no statistical significance. In the experimental group, the total dosage of propofol and sufentanil increased, the microscopic examination time shortened, the choking reaction decreased significantly(*P* < 0.05), and the total incidence of hypoxemia decreased (*P* < 0.05).

**Conclusion:**

The application of nasal high-flow humidification oxygen therapy in elderly painless fiberoptic bronchoscopy improves the oxygenation ability of patients increases the use of narcotic drugs, does not have a great impact on hemodynamics, reduces choking reaction, and is more conducive to the operation of endoscopy doctors.

## 1 Introduction

Fiberoptic bronchoscopy (hereinafter referred to as fiberoptic bronchoscopy) is an important means in the diagnosis, treatment, and rescue of lung diseases, but in practical operation, choking and cardiovascular adverse events will occur in serious cases [1,2,3]. To eliminate the pain of patients during the examination process and facilitate surgical operations, clinical usually use nasal catheter oxygen venous general anesthesia, however, elderly patients are the main population of bronchoscopy, usually chest wall compliance and oxygen reserve function are poor, higher incidence of hypoxemia, considering the need to keep patients spontaneous breathing, anesthesia is more cautious, intraoperative still prone to cough. As a new respiratory support technology, high-flow nasal cannula oxygen therapy (HFNC) has saved countless lives during COVID-19 due to its ability to improve respiratory function and good tolerance [4]. Previous studies have shown that this technique can be used for painless fiber microscopy, improve patient oxygenation, and effectively prevent hypoxic [5]. However, there are few clinical studies of HFNC to reduce the cough response in fiber microscopy. The main purpose of this study is to investigate the effect of HFNC on the postoperative cough response and its related respiratory and hemodynamic effects in elderly patients.

## 2 Methods and materials

### 2.1 General information

This is a prospective, randomized, and controlled study. From June 2021 to December 2021, 130 patients with painless bronchoscopy who were treated in Wuhan No. 1 Hospital were randomly divided into the nasal catheter oxygen group (control group, 65 cases) and the high flow oxygen therapy group (experimental group, 65 cases) by computer-generated random numbers. Each group had 1–2 cases of laryngeal mask into general anesthesia. Finally, 126 cases were enrolled, including 62 cases in the experimental group and 64 cases in the control group.

Patients in the experimental group received HFNC and those in the control group received standard care using. There were no significant differences in age, gender, body mass index (BMI) values, and American Society of Anesthesiologists (ASA) grade between the two groups (*P* > 0.05, Table 1).

**Table 1:** Comparison of general data of patients

Groups	Age (years)	Gender	BMI (kg/m^2^)	ASA grading
		Male/female		I/II/III
Experimental group (*n* = 64)	69.4 ± 12.5	34/31	22.8 ± 2.3	11/35/19
Control group (*n* = 62)	71.9 ± 15.3	31/28	22.5 ± 2.5	10/26/23
*F*/*χ* ^2^ value	2.132	0.210*	0.121	0.462*
*P* value	0.250	0.652	0.645	0.110

Note: * is a *χ* ^2^ value.

Inclusion criteria are as follows: (1) patients aged 65–80 years; (2) patients who met ASA grade I–III; (3) no oxygen saturation 90%; (4) BMI at 18.5–26.5 kg/m^2^; and (5) fiberoptic bronchoscopy + alveolar lavage. Exclusion criteria are as follows: (1) coagulopathy or epistaxis tendency; (2) onset/exacerbation of congestive heart failure; (3) severe aortic stenosis or mitral stenosis; (4) airway obstruction; and (5) requiring endoscopic treatment.

### 2.2 Study methods

Before surgery, all patients need to fast for 8 h and water deprivation for 4 h. The day before microscopy, each patient was evaluated with anesthesia, focusing on the evaluation of cardiopulmonary function and difficult airway prediction. Peripheral venous channels were opened after home entry, and heart rate (HR), blood pressure (BP), and percutaneous pulse oxygen saturation (SpO_2_) were monitored and recorded.

To analyze HR, BP, and peripheral pulse SpO_2_, the following methods are typically employed:

(1) HR
  HR is measured using an HR monitor or electrocardiogram. Counting the number of heartbeats per minute,which can be manually counted by palpating the pulse and counting beats over a specific period, manually counted by palpating the pulse and counting beats over a specific period, typically 15 or 30 s, and then multiplying to obtain beats per minute (bpm).
(2) BP
  BP is measured using a sphygmomanometer (manual or automated) to assess systolic and diastolic pressures: systolic pressure: the pressure in the arteries when the heart contracts and pumps blood and diastolic pressure: the pressure in the arteries when the heart rests between beats.
(3) Peripheral pulse SpO_2_

SpO_2_ is measured using a pulse oximeter, which non-invasively monitors oxygen saturation levels in the blood. A clip-like device is attached to a fingertip or earlobe, emitting light wavelengths through the skin to detect oxygen saturation by measuring the absorption of light. Results are displayed as a percentage of oxygen saturation in the arterial blood (normal values typically range from 95 to 100%).

### 2.3 The experimental group

In the experimental use of HFNC, it is indeed necessary to establish specific optimal dosage selection criteria to ensure treatment efficacy and minimize adverse patient reactions. High-flow humidified oxygen is administered via nasal cannula, with an inspired oxygen concentration of 100% and gas temperature maintained at 37°C. Patients were performed a single intravenous injection of propofol for anesthesia induction. The dose of propofol was calculated by body weight. Anesthesia induction begins with an initial oxygen flow rate of 15 L/min and pre-oxygenation for 1 min before sleep induction. Subsequently, slow intravenous pushes of 5 μg of sufentanil and 1–2 mg/kg of propofol are administered. Patient consciousness is continuously monitored, with medication cessation upon the disappearance of eyelash reflex, using the Ramsay Sedation Scale (RSS) to assess sedation depth. The oxygen flow rate is adjusted to 30–60 mL/min when the RSS score exceeds 4 (delayed or no response to eyebrow tapping or loud auditory stimulus), depending on the patient’s condition. Endoscopic doctors perform two airway surface anesthesia through the biopsy channel of the fiberoptic bronchoscope. For the first time on the glottis, the fiberoptic bronchoscope reached the glottis and injected 2% lidocaine 2 mg/kg into the glottis. After 2–3 min, the fiber bronchoscope passed through the glottis to the upper position of the carina, and then 2% lidocaine 2 mg/kg was injected, and the fiber bronchoscope was removed to the outside of the glottis. After 2–3 min, the surface anesthesia took effect and the operation was continued. When SpO_2_ was less than 90%, the patients were treated with jaw support. When the SpO_2_ was less than 80%, the endoscopist was asked to stop withdrawing from the fiberoptic bronchoscopy, and the mask ventilator was given to assist ventilation. When the SpO_2_ was higher than 90%, the endoscopy was continued. Vital signs are closely monitored throughout the bronchoscopy. If HR drops below 50 beats/min, atropine 0.5 mg is administered intravenously; if mean arterial pressure (MAP) increases by more than 20% from baseline, urapidil 10 mg is administered; if MAP decreases by more than 20% from baseline, ephedrine 6 mg is given intravenously. In case of patient movement or severe coughing during the procedure, sufentanil 2.5–5 μg or propofol 0.2–0.5 mg/kg is administered [6].

After the examination, the patient was sent to the resuscitation room for observation and returned to the ward with stable vital signs and no discomfort. Nasal pain, sore throat, headache, and barotrauma (e.g., pneumothorax and subcutaneous emphysema) were recorded at 1 and 30 min after anesthesia recovery, which was the most common adverse event at 1 min after anesthesia recovery with an incidence of 1.7%, which was not observed at 30 min after anesthesia recovery.

The parameter settings of HFNC were as follows. oxygen concentration, 21–100%; temperature, 31–37°C, and flow rate, up to 70 L/min. The initial setting parameters were oxygen concentration at 80%, temperature at 34°C, and flow rate at 15 L/min. Oxygen concentration and oxygen flow can be adjusted according to the patient’s oxygenation status. HFNC is mainly used to reduce the incidence of intraoperative hypoxemia and cough. The upper limit of oxygen concentration was 100% and the upper limit of flow rate was 40 min/L. If hypoxemia cannot be solved, we will use anesthesia machine-assisted ventilation. However, the oxygen flow rate was usually 40 min/L and the oxygen concentration was 100%.

### 2.4 Control group

Patients were performed a single intravenous injection of propofol for anesthesia induction. The RSS was used to assess sedation depth. The dose of propofol was calculated by body weight. Conventional nasal catheter received oxygen, oxygen flow of 3–6 L/min, and handled respiratory and circulation problems and other experimental groups during the examination.

### 2.5 Observed indicators

MAP, HR, and SpO_2_ were recorded before anesthesia (T0), penetration into the glottis (T1), at (T2), and end of microscopy (T3). The examination time, awakening time, total dosage of propofol and sufentanil, cough reaction, and intraoperative hypoxemia were observed in each group.

Choke score is as follows: 0 no cough; 1 is mild cough, cough no more than 2 times; 2 moderate cough, cough 3–5 times; 3 severe cough, cough >5 [7]. Perioperative hypoxemia classification is as follows: airway opening with mild respiratory depression (90% SpO_2_ < 95%); hypoxia (75% SpO_2_ < 90%, < 60 s) brings the oxygen flow to the airway by mandibular lift; mask ventilation [8] for severe hypoxia (SpO_2_ < 75% or SpO_2_ < 90% = 60 s); if this severe hypoxia cannot be corrected by mask ventilation, a double-tube laryngeal mask is required.

### 2.6 Statistical analysis

Data processing was performed by SPSS20.0 statistical software. Data were expressed as means ± standard deviation, and paired *t*-tests by repeated measurement were used for comparison between the two groups. Count data were described by composition ratio or rate (%), and the *χ* ^2^ test was used for comparison between the two groups. *P* < 0.05 was considered to be statistically significant.


**Informed consent:** All patients were informed and signed informed consent voluntarily. The written consent was received from all participants.
**Ethical approval:** This study was approved by the Ethics Committee of Shenzhen People’s Hospital and complied with the guidelines outlined in the Declaration of Helsinki were followed. This study was approved by the Ethics Committee of Wuhan No. 1 Hospital (Approval No. 2021118).

## 3 Results

### 3.1 Comparison of the perioperative hemodynamic indicators

Compared with the control group, there was no significant difference in MAP and HR at each time point in the experimental group (*P* < 0.05) and no significant difference in oxygen saturation at T0, T4, T1, T2, and T3 in the experimental group (*P* > 0.05); HR and SpO_2_ at different time points, HR and SpO_2_ showed little fluctuation and high stability, as shown in Table 2.

**Table 2:** Changes in vital signs in two groups per time point (*x̅* ± *s*)

Items	Group	T0	T1	T2	T3	*F*	*P*
MAP (mmHg)	Experimental group	99.63 ± 12.25	85.10 ± 9.56	83.25 ± 8.95	88.12 ± 9.50	23.250	0.525
	Control group	97.90 ± 10.35	88.20 ± 10.15	86.28 ± 8.80	90.25 ± 10.36	27.360	0.256
	*F* value	1.56	0.24	0.09	0.35		
	*P* value	0.165	0.859	0.920	0.752		
HR (bpm)	Experimental group	78.25 ± 10.12	81.25 ± 15.37	84.15 ± 14.23	85.32 ± 13.20	5.800	0.450
	Control group	79.10 ± 9.56	82.39 ± 13.25	91.03 ± 15.21	86.12 ± 14.21	12.145	0.024
	*F* value	1.06	0.48	0.23	1.25		
	*P* value	0.355	0.623	0.895	0.256		
SPO_2_ (%)	Experimental group	99.21 ± 1.05	96.15 ± 13.10	96.88 ± 9.50	97.12 ± 3.25	7.245	0.650
	Control group	99.63 ± 12.25	85.10 ± 9.56	83.25 ± 8.95	96.12 ± 9.50	1.250	0.000
	*F* value	2.650	1.320	1.855	2.345		
	*P* value	0.568	0.025	0.015	0.680		

### 3.2 Comparison of microscopy time, awakening time, anesthetic dosage, and cough reaction in the two groups

Compared with the control group, the amount of propofol was increased (*P* < 0.05), and the awakening time was slightly longer, but not statistically significant; the microscopic time was significantly shortened (*P* < 0.05), and the cough response was significantly reduced (*P* < 0.05), as shown in Table 3.

**Table 3:** Comparison of microscopy time, awakening time, amount of anesthesia used, and cough response between the two groups

Groups	Microscopy time (min)	Wake-up time (s)	Total propofol dosage (mg)	Total amount of sufentanil administered (μg)	Choke cough reaction
Experimental group (*n* = 64)	10.04 ± 7.32	2.98 ± 0.85	125.10 ± 54.32	7.52 ± 1.30	1.05 ± 0.35
Control group (*n* = 62)	13.23 ± 9.25	2.25 ± 0.55	102.21 ± 47.20	5.10 ± 1.12	2.29 ± 0.62
*F*/*χ* ^2^ value	2.132	1.350	3.124	6.236	7.457*
*P* value	0.023	1.205	0.000	0.018	0.025

*χ ^2^ value.

### 3.3 Patient occurrence of hypoxemia

In the experimental group, there were 4 cases of mild respiratory depression, significantly fewer than the 10 cases in the control group. The number of patients with oxygen deficiency was two cases in the experimental group, markedly lower than the six cases in the control group. There was one case of malhypoxia in the experimental group, noticeably fewer than the three cases in the control group. The overall incidence of hypoxemia in the experimental group was 7 cases, significantly lower than the 19 cases in the control group. Overall analysis indicated that the incidence of adverse events was significantly lower in the HFNC oxygen group compared to the control group (*P* < 0.05), as depicted in Table 4.

**Table 4:** Development of hypoxemia in both groups (case (%))

Groups	*n*	Mild respiratory depression	Oxygen lack	Malhypoxia	Overall incidence of hypoxemia
Experimental group (*n* = 64)	64	4 (64)	2 (64)	1 (64)	7 (64)
Control group (*n* = 62)	62	10 (62)	6 (62)	3 (62)	19 (62)
*χ* ^2^ value					32.15
*P* value					0.000

Comparison with the control group: *P* < 0.05.

## 4 Discussion

The painless technique is to keep the patients in a quiet and painless state, which greatly reduces their anxiety and fear and improves their medical experience. Meanwhile, it provides operators with better vision and observes the condition more carefully and clearly [9]. However, painless fiberscope also has limitations. Because the respiratory tract has a strong function of defense and rejection, during the operation of invasion, patients are also prone to cough, larynobronchial spasm, and hemodynamic fluctuations, leading to airway damage, hypoxemia, and even cardiac arrest and life-threatening. During general anesthesia of the nasal catheter, the patient must breathe spontaneously. During anesthesia, severe choking due to excessive anesthesia, or the anesthesia depth is too deep, resulting in hypoxemia, which affects the surgical operation.

Fibroscopy is mainly in elderly patients with chronic obstructive emphysema, changes in the physiological structure of the respiratory system, oxygen intake decreased, lung reserve decreased, functional residual volume decreased, and hypoxemia is more frequently seen in general anesthesia [10]. Transnasal high-flow humidification oxygen therapy refers to treatment through high flow special nasal congestion providing patients with regulated and relatively constant oxygen concentration (21–100%), temperature (31–37°C), high flow (60 L/min), and humidity, with higher comfort and patient tolerance than ordinary oxygen therapy [11,12,13]. By providing high-flow oxygen, HFNC can rapidly flush carbon dioxide in the nasopharyngeal cavity, generating positive airway pressure (3–7 cm H_2_O) and increasing end-expiratory lung volume. High-flow ventilation allows the anesthesiologist to leave the patient’s head and avoid obstructing the examiner’s procedure. The application of this technique gives solutions to the contradictions between microscopy operation, adequate oxygen administration, and anesthesia depth control. HFNC-supportive oxygen therapy reduced the incidence of subclinical respiratory depression from 16.3 to 1.6% (*P* < 0.001) [14].

In this study, after anesthesia administration, when the fiber microscope entered the glottis, the experimental group had higher SpO_2_ than the control group, which greatly reduced the incidence of hypoxia, considering that high-flow wet oxygen therapy can maintain a certain level of PEEP, achieve airway opening, reduce dead space, and improve patient ventilation. Oxygen saturation was relatively high in the experimental group, so anesthesiologists were more likely to increase the dose of anesthetic sedation drugs. The study found that the total amount of propofol and sufentanyl in the experimental group was higher than that in the control group, because the anesthesiologist was more likely to increase the dose of anesthetic drugs when using HFNC, and the increased depth of sedation and analgesia reduced the adverse reactions such as cough and body movement [15,16]. Microscopy doctors can observe the lesions more carefully and clearly. Due to the high nasal flow, the number of jaw holding times is significantly reduced, which to some extent liberates the hands of the anesthesiologist and pays more attention to the circulation management of elderly patients. The experimental group reduced the discomfort caused by the high-speed gas flow [17,18], with only very mild adverse events, dry myalgia/nasal pain was the most common adverse event, occurred in only 1.7% of patients, without additional medical care, and disappeared within minutes after recovery from anesthesia. The awakening time was slightly longer, but it did not affect the surgical turnover speed, including the faster metabolism of propofol or sufentanyl due to the short examination time.

HFNC effectively reduces coughing response. The potential mechanisms of HFNC in reducing cough reflexes are as follows: (1) airway conditioning: HFNC provides heated and humidified air, which may reduce airway irritation and sensitivity; (2) washout of respiratory dead space: HFNC can clear carbon dioxide from the upper airways, potentially decreasing respiratory drive and reflex cough; (3) positive airway pressure effects; HFNC delivers a positive airway pressure that may stabilize airway structures and reduce the urge to cough; and (4) decreased irritant exposure: by delivering a high flow of oxygen, HFNC dilutes and washes away airborne irritants, reducing the likelihood of triggering a cough reflex. Moreover, for the FOB deep sedation regimen of midazolam combined with high-dose alfentanil, HFNC oxygen therapy with a higher flow rate (45 or 65 L/min) can significantly reduce the incidence of hypoxemia and cough VAS score and has good antitussive and anti-stress effects [19]. In addition, the proportion of patients who were willing to undergo FOB again was more than 90%. It is suggested that high-flow HFNC can effectively improve the safety and efficacy of FOB under deep sedation. The mechanism may be related to the fact that anesthesiologists are more likely to increase the dose of anesthetic drugs to put patients in deep anesthesia. The increase in the depth of sedation and analgesia reduced adverse reactions such as cough and body movement.

In this experiment, HFNC used 15–60 L/min, mainly considering the guidelines recommended for HFNC to overcome the respiratory pipeline resistance, and the minimum oxygen flow was not less than 15 L/min [11]. However, from the perspective of saving oxygen sources, the next step should be further to find the most appropriate oxygen flow suitable for painless fiber microscopy. HFNC has been widely used in bronchoscopy under light sedation. The application of HFNC under deep sedation is still under exploration. At present, the recommended optimal flow range of HFNC is 45 min/L, which is suitable for most patients with alveolar lavage. However, when patients have severe type II respiratory failure, it is recommended to reduce the oxygen concentration of HFNC to 40–50%.

## 5 Conclusion

The use of nasal high-flow humidification oxygen therapy for painless fiber microscopy in the elderly improves the oxygenation of patients, increases the use of anesthetic drugs, does not have a great impact on hemodynamics, reduces the cough reaction, and is more conducive to the operation of microscopic doctors.
